# A Highly Conserved GEQYQQLR Epitope Has Been Identified in the Nucleoprotein of Ebola Virus by Using an In Silico Approach

**DOI:** 10.1155/2015/278197

**Published:** 2015-02-01

**Authors:** Mohammad Tuhin Ali, Md Ohedul Islam

**Affiliations:** Department of Biochemistry and Molecular Biology, University of Dhaka, Dhaka 1000, Bangladesh

## Abstract

Ebola virus (EBOV) is a deadly virus that has caused several fatal outbreaks. Recently it caused another outbreak and resulted in thousands afflicted cases. Effective and approved vaccine or therapeutic treatment against this virus is still absent. In this study, we aimed to predict B-cell epitopes from several EBOV encoded proteins which may aid in developing new antibody-based therapeutics or viral antigen detection method against this virus. Multiple sequence alignment (MSA) was performed for the identification of conserved region among glycoprotein (GP), nucleoprotein (NP), and viral structural proteins (VP40, VP35, and VP24) of EBOV. Next, different consensus immunogenic and conserved sites were predicted from the conserved region(s) using various computational tools which are available in Immune Epitope Database (IEDB). Among GP, NP, VP40, VP35, and VP30 protein, only NP gave a 100% conserved GEQYQQLR B-cell epitope that fulfills the ideal features of an effective B-cell epitope and could lead a way in the milieu of Ebola treatment. However, successful in vivo and in vitro studies are prerequisite to determine the actual potency of our predicted epitope and establishing it as a preventing medication against all the fatal strains of EBOV.

## 1. Introduction

EBOV is a major member of the viral family Filoviridae and is known to be the highly lethal pathogen responsible for hemorrhagic fever [1]. According to Centers for Disease Control and Prevention (CDC), the disease symptoms include fever (greater than 101.5°F), unexplained hemorrhage (bleeding or bruising), muscle pain, abdominal (stomach) pain, severe headache, vomiting, and diarrhea (http://www.cdc.gov/vhf/ebola/symptoms/).

EBOV genome is composed of linearly arranged genes on a single negative-stranded RNA molecule that encodes the seven structural proteins (NP-VP35-VP40-GP-VP30-VP24-L), where NP, VP, GP, and L stand for nucleoprotein, viral structural protein, glycoprotein, and RNA dependent RNA polymerase, respectively [2]. EBOV is comprised of 5 distinct species: Bundibugyo, Zaire, Reston, Sudan, and Taï Forest. Among them, the Reston species is not known to cause disease in humans, but the fatality rates in outbreaks of the other four species have ranged from 25 to 90% [3].

Now in 2014 West Africa is experiencing the largest outbreak of Ebola, which is due to the Zaire species and is affecting Guinea, Sierra Leone, Liberia, Senegal, and Nigeria [4]. According to World Health Organization (WHO), as of December 31, 2014, a total number of 20,206 EBOV disease cases and 7905 deaths have been reported in the current outbreak (http://www.who.int/csr/disease/ebola/situation-reports/en/).

As there is currently no proven therapeutic solution or vaccination against EBOV and the outbreaks of EBOV have been reported frequently, thus identification of therapeutics is a high priority (http://www.who.int/mediacentre/factsheets/fs103/en/). In addition to this, rapid and reliable Ebola virus specific assays are required for diagnosis and outbreak control. The availability of a great number of sequence information has made the potential B- and T-cell epitope identification an auspicious approach for developing therapeutics and vaccine against infectious disease. Nowadays the use of computational methods has made it easy to predict the epitopes and design vaccine in terms of time and cost. Computer aided vaccine design has been proved as promising approach for combating diseases such as malaria, tumors, and multiple sclerosis [5–7].

In this investigation, we have reported a highly conserved B-cell epitope GEQYQQLR in nucleoprotein of EBOV for the prevention of all the fatal strains of this virus. This approach was accomplished by using bioinformatics analysis of viral structural proteins to find the conserved peptide region to identify a highly immunogenic, accessible, and conserved epitope.

## 2. Materials and Methods

**Figure 1:**
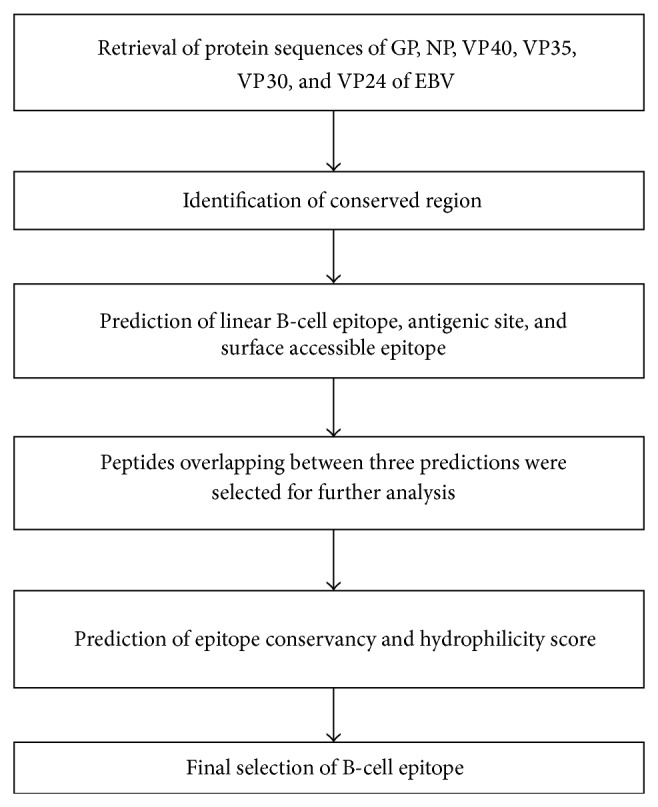
Flowchart summarizing the methodology using a computational approach.

The flowchart in Figure 1 summarizes the steps which have been followed in this study.

### 2.1. Retrieval of Protein Sequences

A sum of 156, 106, 126, 98, 50, and 121 primary amino acid sequences of EBOV envelope glycoprotein (GP), nucleoprotein (NP), matrix protein (VP40), polymerase cofactor (VP35), transcription activator (VP30), and secondary matrix protein (VP24) were retrieved, respectively, from the NCBI protein database (http://www.ncbi.nlm.nih.gov/protein/). These sequences belong to four types of EBOV strain named Zaire, Sudan, Bundibugyo, and Tai forest.

### 2.2. Multiple Sequence Alignment

Multiple sequence alignment of the retrieved protein sequences were performed by using EBI-Clustal Omega program (http://www.ebi.ac.uk/Tools/msa/clustalo/) [8, 9]. Protein sequences that cover highest number of similar and identical amino acids without gap were chosen as the conserved region. Later on, the conserved region was used to predict linear B-cell epitopes, antigenic sites, and surface accessible epitopes.

### 2.3. Prediction of Linear B-Cell Epitope and Antigenic Site

BepiPred (v1.0) was employed to predict linear B-cell epitopes from the conserved region based on hidden Markov model with a default threshold of 0.35 [10]. Besides, the B-cell epitope prediction tool of the immune epitope database (IEDB) (http://tools.immuneepitope.org/bcell/) was used to predict antigenic sites [11]. Here, the Kolaskar and Tongaonkar antigenicity method was applied while predicting the antigenic sites with a default threshold value of 1.0 [12].

### 2.4. Surface Accessible Epitope

Emini surface accessibility prediction tool of the IEDB database (http://tools.immuneepitope.org/tools/bcell/iedb_input) was used to predict the surface accessible epitopes from the conserved region keeping the default threshold value 1.0 unchanged [13].

### 2.5. Prediction of Epitope Conservancy

To predict the conservancy of the candidate epitopes, the IEDB epitope conservancy prediction tool (http://tools.immuneepitope.org/tools/conservancy/iedb_in put) was used [14]. It measures the epitope conservancy based on the sequence identity between epitopes and the given protein sequences.

### 2.6. Prediction of Epitope Hydrophilicity

For the determination of the hydrophilicity of the conserved region, Parker hydrophilicity prediction tool of the IEDB database (http://tools.immuneepitope.org/bcell/) was employed [15]. The default threshold value of 1.571 was used in this study.

### 2.7. Prediction and Structure Analysis of NP

As there was no experimental 3D structure of NP present in PDB database, therefore the 3D structure of Ebola NP was constructed using Phyre2 (Protein Homology/analogY Recognition Engine V 2.0) (http://www.sbg.bio.ic.ac.uk/phyre2/html/) [16]. The Ramachandran plot for our designed 3D structure was done by employing RAMPAGE (http://mordred.bioc.cam.ac.uk/~rapper/rampage.php) to determine the quality and validity of the modeled structure [17]. Later on, the conserved epitope was mapped on the modeled structure of the NP protein by using PyMOL molecular graphics software (version 1.5.0.3) [18].

### 2.8. Conservancy of the Identified Epitope in the Marburg Virus (MBG) NP Protein

A total of 42 primary amino acid sequences of MBG NP were retrieved from NCBI protein database (http://www.ncbi.nlm.nih.gov/protein/). Multiple sequence alignment of the retrieved sequences including one Ebola NP protein was conducted by using EBI-Clustal Omega program (http://www.ebi.ac.uk/Tools/msa/clustalo/) to identify whether the identified epitope is conserved within MBG or not [8, 9].

## 3. Results

### 3.1. Multiple Sequence Alignment

The presence of conserve region has been identified from all the proteins (GP, NP, VP40, VP35, and VP30) except VP24 (Supplementary File 1 to Supplementary File 6 in Supplementary Material available online at http://dx.doi.org/10.1155/2015/278197). The identified conserve regions with their respective primary amino acid sequence are summarized in Table 1.

### 3.2. Prediction and Selection of B-Cell Epitopes

A peptide must ensure the immunogenic and antigenic property to be considered as potential B-cell epitope. Additionally, in antibody mediated immune response, the binding of an antibody with a surface accessible antigenic epitope is important. Therefore, surface accessibility is a prerequisite characteristic for a peptide to become a potential B-cell epitope [19]. By considering these conditions, we have aimed to predict and select B-cell epitopes from the identified conserved sequences of the proteins of EBOV by using various computational tools.

#### 3.2.1. Glycoprotein (GP)

Predicted linear B-cell epitopes, antigenic sites, and surface accessible epitopes from the conserved sequence of GP protein are summarized in Table 2. We observed that only one predicted linear B-cell epitope from this region is HDWTKN. This epitope was found to be overlapped with another surface accessible epitope PHDWTKNITDKI but showed no antigenicity in the Kolaskar and Tongaonkar prediction. Hence, it can be said that none of the predicted epitopes from the conserved region of GP protein fulfilled the criteria to become a potential B-cell epitope.

#### 3.2.2. Nucleoprotein (NP)

A total of ten linear B-cell epitopes, ten antigenic sites, and five surface accessible epitopes have been predicted from the conserved region of NP protein (Table 3). Among them, GEQYQQLR was the only one to satisfy the immunogenic, antigenic, and surface accessible criteria.

#### 3.2.3. Matrix Protein (VP40) and Transcription Factor (VP30)

The predicted linear B-cell epitopes, antigenic sites, and surface accessible epitopes from the conserved sequences of VP40 and VP30 proteins are summarized in Tables 4 and 5, respectively. Here, none of the predicted peptides overlaps among these three predictions.

#### 3.2.4. Polymerase Cofactor (VP35)

In case of VP35 protein, a total of two linear B-cell epitopes, two antigenic sites, and one surface accessible epitope were predicted (Table 6). Only PVPPSP epitope from the conserve sequence of VP35 was found to be overlap among all three predictions.

### 3.3. Conservancy Analysis

**Figure 2:**
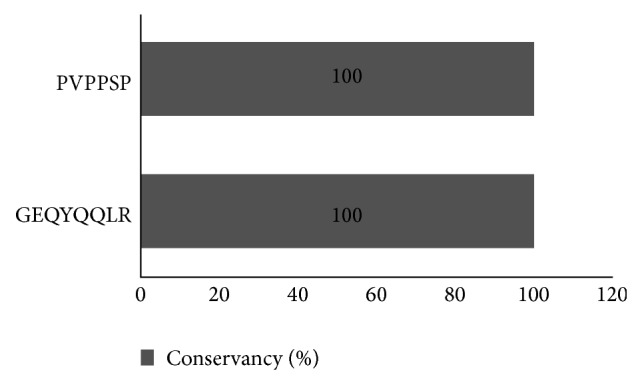
Illustration of the results of epitope conservancy analysis.

GEQYQQLR and PVPPSP showed 100% conservancy among all the fatal strains of EBOV when all GP and VP35 protein sequences were implemented for conservancy analysis (Figure 2).

### 3.4. GEQYQQLR Epitope Demonstrated Hydrophilicity

**Figure 3:**
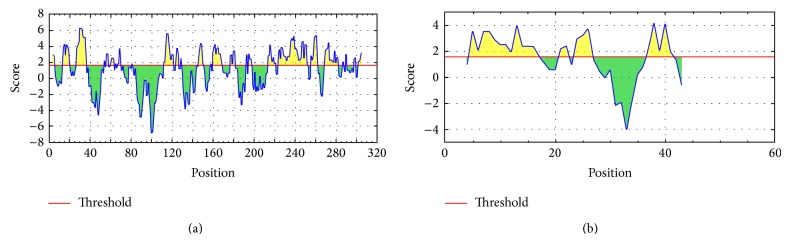
Hydrophilicity plot of the conserve sequences of NP and VP35 protein. Yellow color represents hydrophilic regions in either graph. Graphs (a) and (b) both present the parker hydrophilicity plot for the conserve region of NP and VP35, respectively. Maximum hydrophilicity was found to be 6.243 and 4.20 for NP and VP35, respectively.

The results of Parker hydrophilicity prediction tool for the conserved sequences of NP and VP35 proteins are given in Figure 3. The average hydrophilicity score of the GP protein was 1.122. Most importantly, our predicted GEQYQQLR epitope (226–233 amino acids position in Figure 3(a)) was found to cross the threshold (1.571) value. On the contrary, PVPPSP epitope (19–24 amino acids position in Figure 3(b)) demonstrated lower hydrophilicity score than the threshold value (1.571). For passing all filter and criteria, GEQYQQLR was selected as the ultimate desired B-cell epitope.

### 3.5. Homology Modeling of Ebola NP and Epitope Mapping

**Figure 4:**
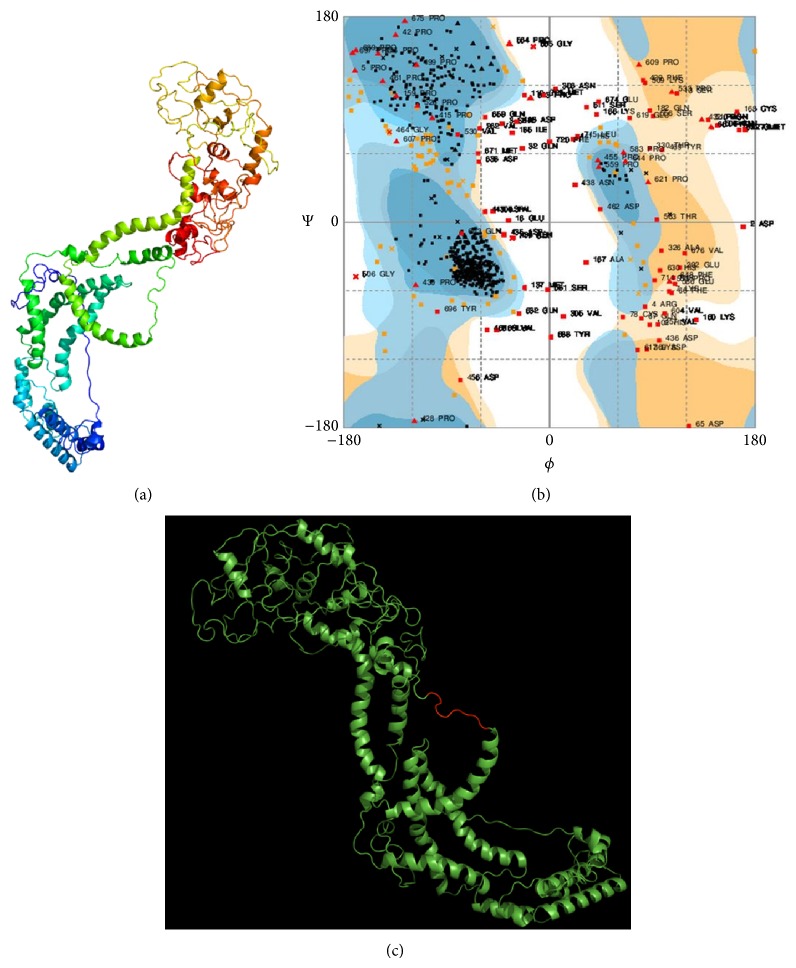
Mapping of B-cell epitope on the surface of modeled Ebola NP. (a) The homology modeled structure of the Ebola NP protein depicted from the Phyre2 result in cartoon format. (b) Ramachandran plot of the designed 3D structure of the Ebola NP which is obtained from RAMPAGE. (c) Mapping of GEQYQQLR epitope on the surface of Ebola NP where the epitope sequence is represented by red color and rest of the protein structure is represented by green color.

The Phyre2 generated 3D structure of Ebola NP is presented in Figure 4(a). From the results of Ramachandran plot analysis (Figure 4(b)), it was observed that 86.3% amino acid residues of the modeled Ebola NP were in the acceptable region of the plot. In Figure 4(c), the mapping of conserved epitope GEQYQQLR on the surface of designed Ebola NP was given.

### 3.6. Sequence Conservancy with Marburg Virus

In Supplementary File 7 (see Supplementary Material), the result of multiple sequence alignment of the nucleoprotein of Marburg and Ebola virus is given. The B-cell epitope GEQYQQLR was found to have 100% sequence conservancy with Marburg NP.

## 4. Discussion

EBOV infection which is endemic in central Africa demonstrates high fatality rate in humans. Application of proper treatment and absence of effective vaccine combined with the lethal effect of EBOV have made this virus an important public health pathogen. Although several reports have been made in recent years for the development of vaccine against EBOV, but till date neither effective vaccines nor drugs are licensed for human use against EBOV [20–22]. As the clinical symptoms at the early stage caused by other pathogens are comparable with that of EBOV hemorrhagic fever, therefore developing rapid, sensitive, reliable, and virus specific diagnostic tests is in urgent need. In this regard, EBOV antigen detection assay using specific monoclonal antibodies (mAb) would be one of the best possible ways to detect viral infection at the early stage in the field setting [23].

In the year 2000 and 2007, identification of the multiple monoclonal antibodies including neutralizing antibodies against EBOV and the epitopes from viral glycoprotein in rodent models was done, respectively [24, 25]. More recently, in 2014, Becquart et al. have identified continuous B-cell epitopes in the GP, NP, VP40, and VP35 proteins of EBOV [26]. In spite of these findings, the information on human B-cell epitopes from the structural proteins of EBOV is not sufficient. In addition to this, the works that have been done on humoral response associated with EBOV were against some specific strains, mostly Zaire. To present dates there is no evidence of work that includes all the fatal strains of EBOV. In this study, we have tried to identify highly conserved B-cell epitopes from GP, NP, VP40, VP35, and VP24 proteins of EBOV.

Results from the multiple sequence alignment have showed that GP, NP, VP40, VP35, and VP30 proteins are less mutation prone than VP24 protein. We did not get any conserved region from VP24 protein. Therefore, it was excluded from further analysis based on identified conserved regions. The remaining conserved regions were analyzed in turns of B-cell antigenicity, surface accessibility, persistent conservancy, and hydrophilicity to select final B-cell epitope. We have observed that none of the predicted epitopes from the conserved region of GP, VP40, VP35, and VP30 proteins possesses these properties. Only in case of NP, we have identified that GEQYQQLR epitope displays all the key properties mentioned above. Furthermore, we performed structural analysis by using a homology modeling and epitope mapping approach to find out whether or not this epitope present on the surface of modeled EBOV NP. In our analysis, GEQYQQLR epitope was found to have the existence on the surface of our modeled EBOV nucleoprotein. Thereby it gives further validation that GEQYQQLR epitope possesses all the key properties to become a successful B-cell epitope.

Conventional molecular immunoassay techniques of virus detection are based on conserved epitopes of specific viral protein. Considering this principle, EBOV NP could be the most important antigenic protein due to its abundance in EBOV in immunological detection [27, 28]. In this study, it has been proved that the identified GEQYQQLR epitope is fully conserved at the C-terminal region of NP in all the EBOV strain included. Although significant variability of the C-terminal region has been reported but our predicted epitope was found to be conserved well [28].

Finally, the result of multiple sequence alignment with MBG and EBOV NP demonstrated that the identified epitope GEQYQQLR has 100% conservancy among the MBG NP. This dictates the importance of designing of a common vaccine against both of the virus using this conserved epitope. In addition to this, it could be an aid to develop new assays of viral antigen detection such as immune chromatography based rapid detection method.

As we have identified this epitope by employing a computational approach, hence determination of the real efficacy, immunogenicity, and stability of this epitope needs to be done by further in vivo and in vitro studies. An adjuvant might be needed to conjugate with this peptide if it shows poor immunogenicity or stability while testing in recipient's body [29].

Generally, development of diagnostic tools, vaccines, and therapeutics against viral diseases is accelerated by the characterization of antigenic sites in the viral proteins [23, 30]. In this study, by employing sequence analysis and computational prediction we have identified GEQYQQLR epitope from EBOV NP as the best B-cell epitope target for designing new mAb based specific rapid detection assays and therapeutics against this EBOV upon successful in vitro and in vivo studies.

## Supplementary Material

Supplementary File 1: Multiple sequence alignment of Envelope Glycoprotein (GP) of EBOV. The conserved region among all the fatal strains of EBOV is highlighted by yellow color. In this figure, a position which have single, fully conserved residues is indicated by an ∗(asterisk). Conservation between groups of strongly similar amino acids and of weakly similar amino acids is indicated by colon (:) and period (.), respectively.
Supplementary File 2: Multiple sequence alignment of Nucleoprotein (NP) of EBOV. File legend as in Supplementary File 1.
Supplementary File 3: Multiple sequence alignment of Matrix protein (VP40) of EBOV. File legend as in Supplementary File 1.
Supplementary File 4: Multiple sequence alignment of Polymerase cofactor (VP35) of EBOV. File legend as in Supplementary File 1.
Supplementary File 5: Multiple sequence alignment of Transcription activator (VP30) of EBOV. File legend as in Supplementary File 1.
Supplementary File 6: Multiple sequence alignment of Secondary matrix protein (VP24) of EBOV. This alignment depicted that the rate of mutation is high in this protein. Therefore, we didn't find any conserved region from VP24 protein. File legend as in Supplementary File 1.
Supplementary File 7: Multiple sequence alignment of Nucleoprotein (NP) of MBG and EBOV. File legend as in Supplementary File 1.

## Figures and Tables

**Table 1:** Conserved regions from proteins identified by EBI Clustal Omega program.

Sequence	Protein	Position
EGLMHNQDGLICGLRQLANETTQALQLFLRATTEL	Glycoprotein (GP)	545–633
RTFSILNRKAIDFLLQRWGGTCHILGPDCCIEPHDW		
TKNITDKIDQIIHDFVDK		

DGVKRLEELLPAVSSGKNIKRTLAAMPEEETTEAN	Nucleoprotein (NP)	112–419
AGQFLSFASLFLPKLVVGEKACLEKVQRQIQVHAE		
QGLIQYPTAWQSVGHMMVIFRLMRTNFLIKFLLIHQ		
GMHMVAGHDANDAVISNSVAQARFSGLLIVKTVL		
DHILQKTERGVRLHPLARTAKVKNEVNSFKAALSS		
LAKHGEYAPFARLLNLSGVNNLEHGLFPQLSAIALG		
VATAHGSTLAGVNVGEQYQQLREAATEAEKQLQQ		
YAESRELDHLGLDDQEKKILMNFHQKKNEISFQQT		
NAMVTLRKERLAKLTEAITAASLPKTSG		

GIPDHPLRLLRIGNQAFLQEFVLPPVQLPQYFTFDLT	Matrix protein (VP40)	141–197
ALKLITQPLPAATWTDDTPT		

IHIRSRGDIPRACQKSLRPVPPSPKIDRGWVCVFQLQ	Polymerase cofactor (VP35)	295–340
DGKTLGLKI		

LTVPPAPKDICPTLKKGFLCDSSFCKKDHQLESLTD	Transcription activator (VP30)	62–111
RELLLLIARKTCGS		

**Table 2:** Linear B-cell epitope, antigenic regions, and surface accessible epitopes predicted from the conserved region of glycoprotein (GP).

Peptide	Length
BepiPred analysis (linear B-cell epitope)	
HDWTKN	6

IEDB analysis (antigenic sites)	
DGLICGLRQL	10
QALQLFLR	8
TFSILNR	7
IDFLLQR	7
TCHILGPDCCIEP	13

Surface accessible epitope	
QLANETTQ	8
ATTELR	6
PHDWTKNITDKI	12

**Table 3:** Linear B-cell epitope, antigenic regions, and surface accessible epitopes predicted from the conserved region of nucleoprotein (NP).

Peptide	Length
BepiPred analysis (linear B-cell epitope)	
SSGKN	5
AMPEEETTEANA	12
TAWQ	4
HDANDAV	7
KVKN	4
KHGEYA	6
AHGS	4
VGEQYQQLREAATEAEKQLQQYAESR	26
LDDQ	4
EISF	4

IEDB analysis (antigenic sites)	
KRLEELLPAVSSG	13
GQFLSFASLFLPKLVVGEKACLEK	55
VQRQIQVHAEQGLIQYPTAWQSV	
GHMMVIFR	
LIKFLLIHQ	9
NDAVISNSVAQA	12
FSGLLIVKTVLDHILQ	16
GVRLHPLARTA	11
SFKAALSSLAKHGEYAPFARLLNLS	25
HGLFPQLSAIALGVATA	17
GSTLAGVNVGEQYQQLRE	18
LDHLGL	6

Surface accessible epitope	
MPEEETTE	8
AKVKNE	6
GEQYQQLR	8
AATEAEKQLQQYAESREL	18
FHQKKNE	7
LRKERL	6

**Table 4:** Linear B-cell epitope, antigenic regions, and surface accessible epitopes predicted from the conserved region of VP40.

Peptide	Length
BepiPred analysis (linear B-cell epitope)	
GIPD	4

IEDB analysis (antigenic sites)	
DHPLRLLRIG	10
QAFLQEFVLPPVQLPQYFTFDLTAL	35
KLITQPLPAA	

Surface accessible epitope	
VQLPQYF	7

**Table 5:** Linear B-cell epitope, antigenic regions, and surface accessible epitopes predicted from the conserved region of VP30.

Peptide	Length
BepiPred analysis (linear B-cell epitope)	
LTVPPAPKDIC	11

IEDB analysis (antigenic sites)	
PPAPKDICPTLKKGFLCDSSFCKKD	25

Surface accessible epitope	
CKKDHQ	6

**Table 6:** Linear B-cell epitope, antigenic regions, and surface accessible epitopes predicted from the conserved region of VP35.

Peptide	Length
BepiPred analysis (linear B-cell epitope)	
GDIPRA	6
SLRPVPPSPKID	12

IEDB analysis (antigenic sites)	
PRACQKSLRPVPPSP	15
GWVCVFQLQ	9

Surface accessible epitope	
PVPPSPKID	9
