# Supplementary figures and images for: A Highly Conserved GEQYQQLR Epitope Has Been Identified in the Nucleoprotein of Ebola Virus by Using an In Silico Approach
Source: Adv Bioinformatics. 2015 Feb 1;2015:278197. doi: 10.1155/2015/278197 (PMC4331325; doi:10.1155/2015/278197)

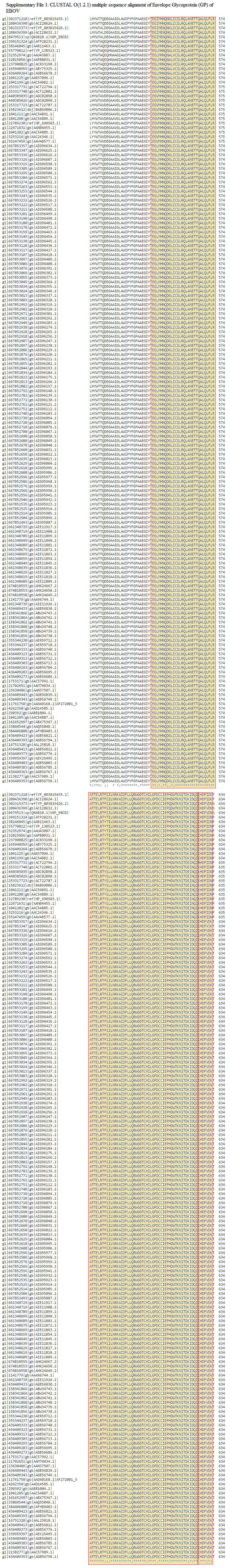

Supplement: Supplementary file 1 — Multiple sequence alignment of Envelope Glycoprotein (GP) of EBOV. The conserved region among all the fatal strains of EBOV is highlighted by yellow color. In this figure, a position which have single, fully conserved residues is indicated by an ∗(asterisk). Conservation between groups of strongly similar amino acids and of weakly similar amino acids is indicated by colon (:) and period (.), respectively. Supplementary File 2: Multiple sequence alignment of Nucleoprotein (NP) of EBOV. File legend as in Supplementary File 1. Supplementary File 3: Multiple sequence alignment of Matrix protein (VP40) of EBOV. File legend as in Supplementary File 1. Supplementary File 4: Multiple sequence alignment of Polymerase cofactor (VP35) of EBOV. File legend as in Supplementary File 1. Supplementary File 5: Multiple sequence alignment of Transcription activator (VP30) of EBOV. File legend as in Supplementary File 1. Supplementary File 6: Multiple sequence alignment of Secondary matrix protein (VP24) of EBOV. This alignment depicted that the rate of mutation is high in this protein. Therefore, we didn't find any conserved region from VP24 protein. File legend as in Supplementary File 1. Supplementary File 7: Multiple sequence alignment of Nucleoprotein (NP) of MBG and EBOV. File legend as in Supplementary File 1. [file 278197.f1.zip › 278197 Supplemental Materials/Supplementary File 1.JPG]

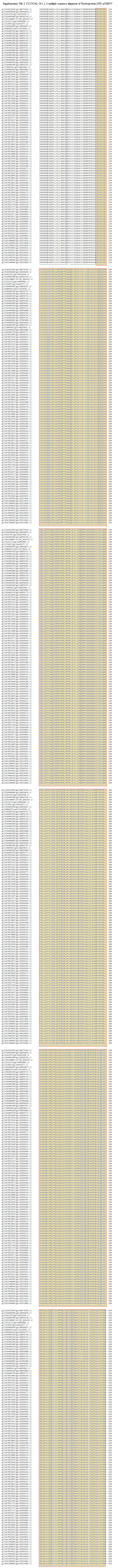

Supplement: Supplementary file 1 — Multiple sequence alignment of Envelope Glycoprotein (GP) of EBOV. The conserved region among all the fatal strains of EBOV is highlighted by yellow color. In this figure, a position which have single, fully conserved residues is indicated by an ∗(asterisk). Conservation between groups of strongly similar amino acids and of weakly similar amino acids is indicated by colon (:) and period (.), respectively. Supplementary File 2: Multiple sequence alignment of Nucleoprotein (NP) of EBOV. File legend as in Supplementary File 1. Supplementary File 3: Multiple sequence alignment of Matrix protein (VP40) of EBOV. File legend as in Supplementary File 1. Supplementary File 4: Multiple sequence alignment of Polymerase cofactor (VP35) of EBOV. File legend as in Supplementary File 1. Supplementary File 5: Multiple sequence alignment of Transcription activator (VP30) of EBOV. File legend as in Supplementary File 1. Supplementary File 6: Multiple sequence alignment of Secondary matrix protein (VP24) of EBOV. This alignment depicted that the rate of mutation is high in this protein. Therefore, we didn't find any conserved region from VP24 protein. File legend as in Supplementary File 1. Supplementary File 7: Multiple sequence alignment of Nucleoprotein (NP) of MBG and EBOV. File legend as in Supplementary File 1. [file 278197.f1.zip › 278197 Supplemental Materials/Supplementary File 2.JPG]

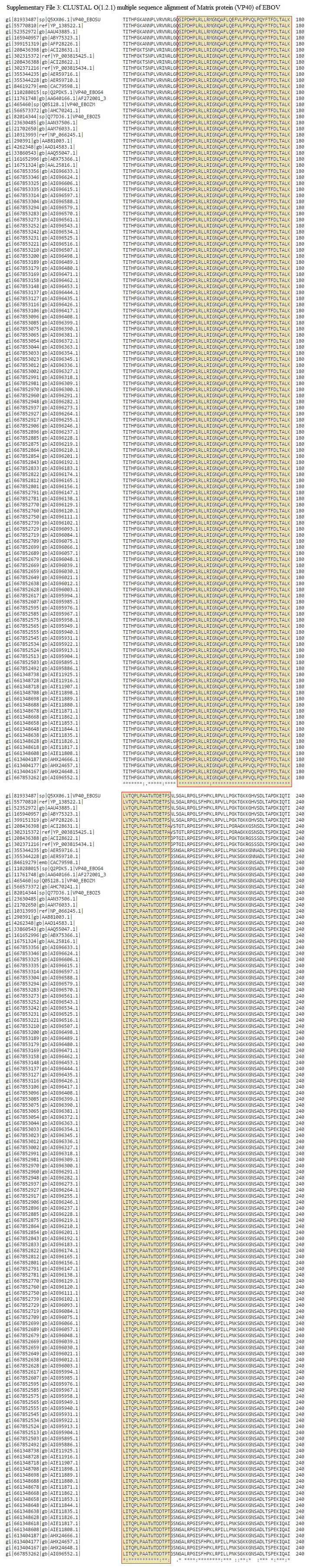

Supplement: Supplementary file 1 — Multiple sequence alignment of Envelope Glycoprotein (GP) of EBOV. The conserved region among all the fatal strains of EBOV is highlighted by yellow color. In this figure, a position which have single, fully conserved residues is indicated by an ∗(asterisk). Conservation between groups of strongly similar amino acids and of weakly similar amino acids is indicated by colon (:) and period (.), respectively. Supplementary File 2: Multiple sequence alignment of Nucleoprotein (NP) of EBOV. File legend as in Supplementary File 1. Supplementary File 3: Multiple sequence alignment of Matrix protein (VP40) of EBOV. File legend as in Supplementary File 1. Supplementary File 4: Multiple sequence alignment of Polymerase cofactor (VP35) of EBOV. File legend as in Supplementary File 1. Supplementary File 5: Multiple sequence alignment of Transcription activator (VP30) of EBOV. File legend as in Supplementary File 1. Supplementary File 6: Multiple sequence alignment of Secondary matrix protein (VP24) of EBOV. This alignment depicted that the rate of mutation is high in this protein. Therefore, we didn't find any conserved region from VP24 protein. File legend as in Supplementary File 1. Supplementary File 7: Multiple sequence alignment of Nucleoprotein (NP) of MBG and EBOV. File legend as in Supplementary File 1. [file 278197.f1.zip › 278197 Supplemental Materials/Supplementary File 3.JPG]

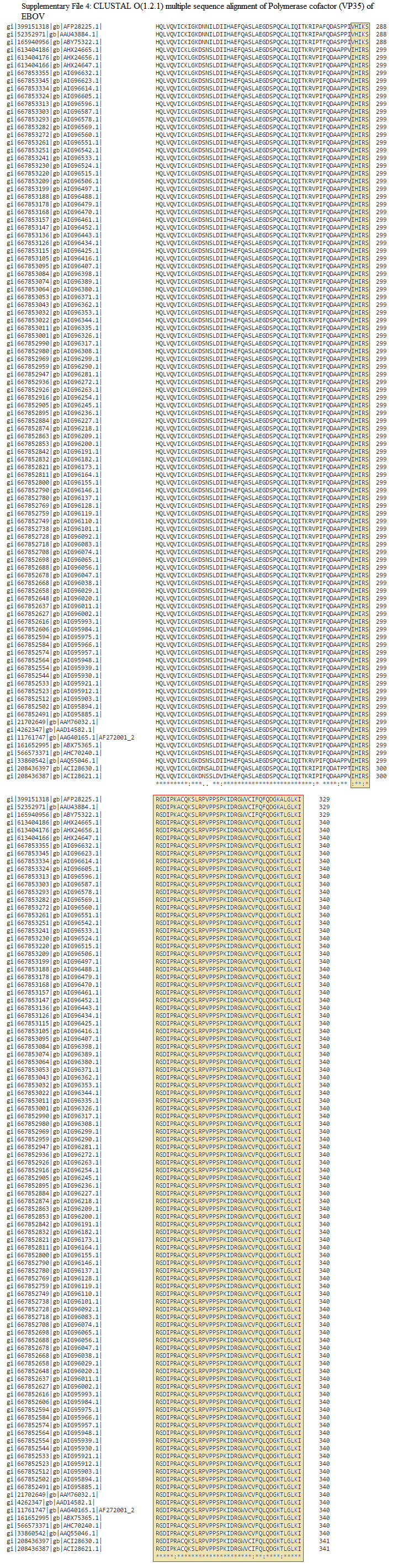

Supplement: Supplementary file 1 — Multiple sequence alignment of Envelope Glycoprotein (GP) of EBOV. The conserved region among all the fatal strains of EBOV is highlighted by yellow color. In this figure, a position which have single, fully conserved residues is indicated by an ∗(asterisk). Conservation between groups of strongly similar amino acids and of weakly similar amino acids is indicated by colon (:) and period (.), respectively. Supplementary File 2: Multiple sequence alignment of Nucleoprotein (NP) of EBOV. File legend as in Supplementary File 1. Supplementary File 3: Multiple sequence alignment of Matrix protein (VP40) of EBOV. File legend as in Supplementary File 1. Supplementary File 4: Multiple sequence alignment of Polymerase cofactor (VP35) of EBOV. File legend as in Supplementary File 1. Supplementary File 5: Multiple sequence alignment of Transcription activator (VP30) of EBOV. File legend as in Supplementary File 1. Supplementary File 6: Multiple sequence alignment of Secondary matrix protein (VP24) of EBOV. This alignment depicted that the rate of mutation is high in this protein. Therefore, we didn't find any conserved region from VP24 protein. File legend as in Supplementary File 1. Supplementary File 7: Multiple sequence alignment of Nucleoprotein (NP) of MBG and EBOV. File legend as in Supplementary File 1. [file 278197.f1.zip › 278197 Supplemental Materials/Supplementary File 4.JPG]

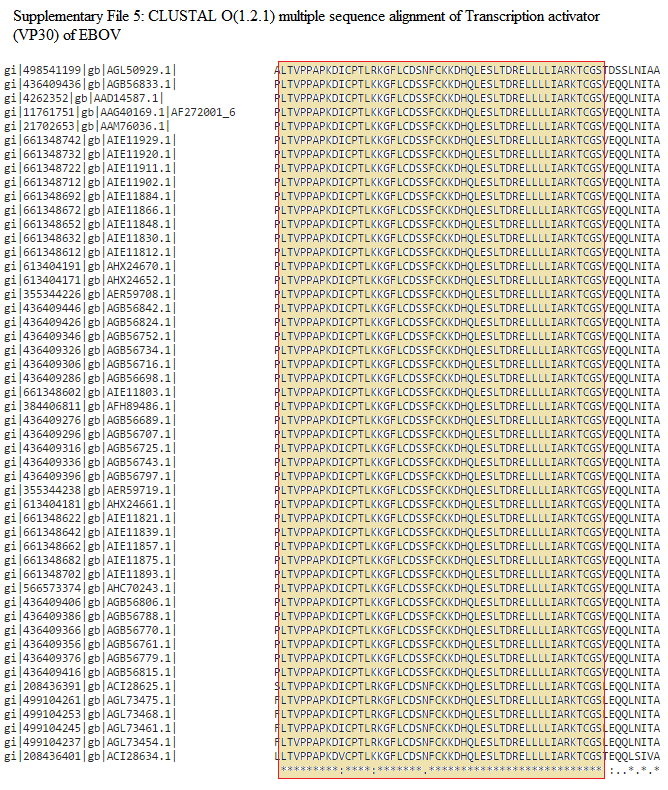

Supplement: Supplementary file 1 — Multiple sequence alignment of Envelope Glycoprotein (GP) of EBOV. The conserved region among all the fatal strains of EBOV is highlighted by yellow color. In this figure, a position which have single, fully conserved residues is indicated by an ∗(asterisk). Conservation between groups of strongly similar amino acids and of weakly similar amino acids is indicated by colon (:) and period (.), respectively. Supplementary File 2: Multiple sequence alignment of Nucleoprotein (NP) of EBOV. File legend as in Supplementary File 1. Supplementary File 3: Multiple sequence alignment of Matrix protein (VP40) of EBOV. File legend as in Supplementary File 1. Supplementary File 4: Multiple sequence alignment of Polymerase cofactor (VP35) of EBOV. File legend as in Supplementary File 1. Supplementary File 5: Multiple sequence alignment of Transcription activator (VP30) of EBOV. File legend as in Supplementary File 1. Supplementary File 6: Multiple sequence alignment of Secondary matrix protein (VP24) of EBOV. This alignment depicted that the rate of mutation is high in this protein. Therefore, we didn't find any conserved region from VP24 protein. File legend as in Supplementary File 1. Supplementary File 7: Multiple sequence alignment of Nucleoprotein (NP) of MBG and EBOV. File legend as in Supplementary File 1. [file 278197.f1.zip › 278197 Supplemental Materials/Supplementary File 5.JPG]

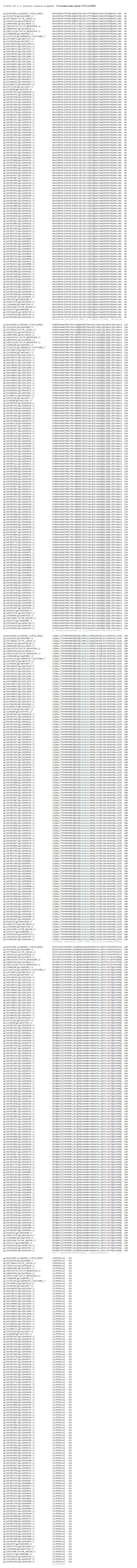

Supplement: Supplementary file 1 — Multiple sequence alignment of Envelope Glycoprotein (GP) of EBOV. The conserved region among all the fatal strains of EBOV is highlighted by yellow color. In this figure, a position which have single, fully conserved residues is indicated by an ∗(asterisk). Conservation between groups of strongly similar amino acids and of weakly similar amino acids is indicated by colon (:) and period (.), respectively. Supplementary File 2: Multiple sequence alignment of Nucleoprotein (NP) of EBOV. File legend as in Supplementary File 1. Supplementary File 3: Multiple sequence alignment of Matrix protein (VP40) of EBOV. File legend as in Supplementary File 1. Supplementary File 4: Multiple sequence alignment of Polymerase cofactor (VP35) of EBOV. File legend as in Supplementary File 1. Supplementary File 5: Multiple sequence alignment of Transcription activator (VP30) of EBOV. File legend as in Supplementary File 1. Supplementary File 6: Multiple sequence alignment of Secondary matrix protein (VP24) of EBOV. This alignment depicted that the rate of mutation is high in this protein. Therefore, we didn't find any conserved region from VP24 protein. File legend as in Supplementary File 1. Supplementary File 7: Multiple sequence alignment of Nucleoprotein (NP) of MBG and EBOV. File legend as in Supplementary File 1. [file 278197.f1.zip › 278197 Supplemental Materials/Supplementary File 6.JPG]

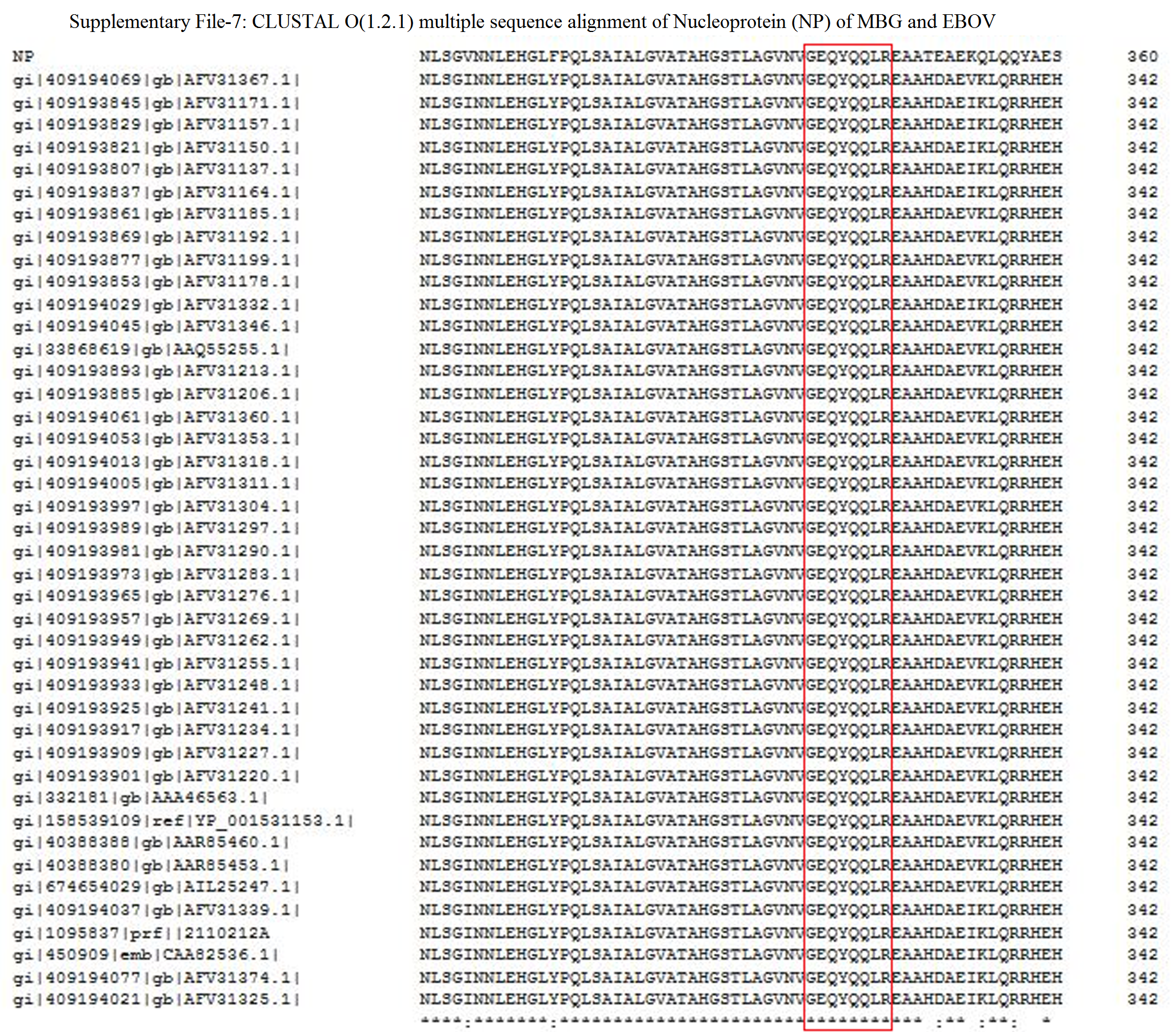

Supplement: Supplementary file 1 — Multiple sequence alignment of Envelope Glycoprotein (GP) of EBOV. The conserved region among all the fatal strains of EBOV is highlighted by yellow color. In this figure, a position which have single, fully conserved residues is indicated by an ∗(asterisk). Conservation between groups of strongly similar amino acids and of weakly similar amino acids is indicated by colon (:) and period (.), respectively. Supplementary File 2: Multiple sequence alignment of Nucleoprotein (NP) of EBOV. File legend as in Supplementary File 1. Supplementary File 3: Multiple sequence alignment of Matrix protein (VP40) of EBOV. File legend as in Supplementary File 1. Supplementary File 4: Multiple sequence alignment of Polymerase cofactor (VP35) of EBOV. File legend as in Supplementary File 1. Supplementary File 5: Multiple sequence alignment of Transcription activator (VP30) of EBOV. File legend as in Supplementary File 1. Supplementary File 6: Multiple sequence alignment of Secondary matrix protein (VP24) of EBOV. This alignment depicted that the rate of mutation is high in this protein. Therefore, we didn't find any conserved region from VP24 protein. File legend as in Supplementary File 1. Supplementary File 7: Multiple sequence alignment of Nucleoprotein (NP) of MBG and EBOV. File legend as in Supplementary File 1. [file 278197.f1.zip › 278197 Supplemental Materials/Supplementary File 7.JPG]
